# Performance of 6 routine coagulation assays on the new Roche Cobas t711 analyzer

**DOI:** 10.1016/j.plabm.2019.e00146

**Published:** 2019-11-09

**Authors:** Marlies Oostendorp, Roefke L. Noorman, J. Dinant Nijenhuis, Jacques B. de Kok

**Affiliations:** a Laboratory of Clinical Chemistry, Deventer Hospital, Deventer, the Netherlands; b Department of Clinical Chemistry and Hematology, Rijnstate Hospital, Arnhem, the Netherlands

**Keywords:** Cobas t711, Coagulation assays, Analytical performance, Unfractionated heparin, Sample stability, aPTT, Activated partial thromboplastin time, PT, Prothrombin time, INR, International normalized ratio, UFH, Unfractionated heparin, LMWH, Low molecular weight heparin

## Abstract

**Objectives:**

For new analyzers or tests, analytical evaluation is required before implementation in the clinical laboratory. We evaluated the novel Roche Cobas t711 analyzer with six newly developed coagulation assays: the activated partial thromboplastin time (aPTT), prothrombin time (PT), international normalized ratio (INR), fibrinogen, d-dimer and anti-Xa. The evaluation included imprecision experiments, method comparison with the currently used Stago STA-R Evolution, monitoring of unfractionated heparin (UFH) with aPTT, a fast centrifugation protocol to improve turn-around time, and determination of sample stability in whole blood and plasma.

**Design and methods:**

Imprecision and method comparison were assessed using commercial quality control samples and patient samples, respectively. For dose monitoring of UFH with the aPTT, samples from patients treated with UFH were used. Samples from healthy volunteers were collected for evaluation of the fast centrifugation protocol (5’ 2750×g) and for investigating sample stability over 6–8 h.

**Results:**

Results for between-run precision were within the desirable specification. Method comparison showed an excellent agreement for fibrinogen, d-dimer and anti-Xa. For aPTT, PT and INR, a good correlation was found, but results were significantly lower on the t711 compared to the STA-R Evolution, which is caused by different coagulation activators. Results from the fast centrifugation protocol differed not significantly from the standard protocol (15’ 2500×g). Blood and plasma samples were stable at room temperature up to 6 and 8 h, respectively.

**Conclusions:**

The t711 coagulation analyzer with 6 novel tests is suitable for routine use in clinical laboratories.

## 1 Introduction

Laboratory accreditation according to the international standard ISO15189:2012 demands that laboratories validate each novel assay and instrument before it can be used in clinical practice [1]. When methods developed by certified *in vitro* diagnostic companies are implemented without any modification, verification (instead of validation) of the method will suffice (ISO15189 paragraph 5.5.1.2). This implies that the laboratory should confirm that the performance characteristics as stated by the manufacturer are met. Although the ISO15189 standard does not specify which performance characteristics need to be evaluated, common verification procedures include at least analysis of imprecision and method comparison [2,3]. Additional performance characteristics can be included in the verification process based on national or local guidelines, or the laboratory specialist’s professional opinion.

In this study, a verification was performed of six coagulations assays on the novel Roche Cobas coagulation analyzer t711, including the activated partial thromboplastin time (aPTT), prothrombin time (PT), international normalized ratio (INR), d-dimer, fibrinogen, and anti-Xa for monitoring of both unfractionated heparin (UFH) and low molecular weight heparin (LMWH) therapy. Results were compared with the corresponding methods on the Stago STA-R Evolution.

The aims of this study were first to reproduce previous results on imprecision and method comparison [[4], [5], [6], [7]], second to evaluate the performance of the anti-Xa assay (not previously published), third to determine whether the aPTT can be used to monitor UFH therapy, fourth to investigate a fast centrifugation protocol in order to shorten turn-around time, and finally to study sample stability over time, since this is known to be method and reagent dependent [8].

## 2 Materials and methods

### 2.1 Analyzers and choice of reagents

The Roche Cobas t711 (Roche Diagnostics GmbH, Mannheim, Germany) is a fully automated, continuous random-access coagulation analyzer. Liquid and lyophilized reagents are available and the latter are automatically reconstituted by the t711. The test menu comprises both general and specialized assays based upon optical clotting, chromogenic and immunoturbidimetric detection. Results were compared with the Stago STA-R Evolution coagulation analyzer (Diagnostica Stago S.A.S., Asnières-sur-Seine, France). An overview of the investigated assays, reagents, detection methods and corresponding reference intervals on both systems is provided in Table 1.

**Table 1:** Overview of the investigated assays, reagents, methods and reference intervals on the Roche Cobas t711 and Stago STA-R Evolution coagulation analyzers.

	Cobas t711			STA-R Evolution		
	Reagent^a^	Method	Ref.^b^	Reagent^a^	Method	Ref.
aPTT (s)	aPTT with ellagic acid activator (liq)	Optical clot detection	22.6–30.6	Cephascreen with polyphenolic activator (liq)	Mechanical clot detection	26–34^c^
PT (s)	PT Rec with recombinant human thromboplastin activator (lyo)	Optical clot detection	8.4–10.6	Neoplastin CI plus with rabbit brain thromboplastin activator (lyo)	Mechanical clot detection	12–15^c^
INR	PT Owren with rabbit brain thromboplastin activator and bovine plasma free of factors II, VII and X (lyo)	Optical clot detection INR calibration with PT Cal Set^e^: MNPT 20.2 s and ISI 0.904.	0.8–1.2^d^	Hepato-Prest with rabbit brain thromboplastin activator and bovine plasma free of factors II, VII and X (lyo)	Mechanical clot detection INR calculation: MNPT 29.8 s and ISI 0.88.	0.8–1.2^d^
D-dimer (μg/L)	Tina-quant D-Dimer Gen. 2 (liq)	Particle-enhanced immuno turbidimetry	<500	Liatest (liq)	Particle-enhanced immuno turbidimetry	<500
Fibrinogen (g/L)	Fibrinogen with bovine thrombin (lyo)	Clauss clotting assay with optical detection	1.9–4.1	Fibrinogen with human thrombin (liq)	Clauss clotting assay with mechanical detection	2.0–4.5
Anti-Xa (IU/mL)	Anti-Xa with bovine factor Xa (liq)	Chromogenic Calibration with Hep Cal Set	NA^f^	Anti-Xa with bovine factor Xa (liq)	Chromogenic Calibration with Multi Hep Calibrator	NA^f^

aPTT: activated partial thromboplastin time. PT: prothrombin time. INR: international normalized ratio. Ref: reference interval. ISI: international sensitivity index. MNPT: mean normal PT. NA: not applicable.

a liq: liquid reagent; lyo: lyophilized reagent.

b As stated by the manufacturer.

c Reference interval based on Toulon et al. [9].

d Therapeutic range 2.0–3.0 or 2.5–3.5, depending on clinical indication.

e PT Owren was calibrated against National Institute for Biological Standards and Contol RBT/05 with tilted tube method according to WHO technical report 889.

f Normal ranges are zero or undetectable. Therapeutic ranges depend on the kind of heparin and dosing regimen.

For determining the INR on the t711, two different reagents are available (PT-rec and PT-Owren) with two modes of INR calculation each: uploading the lot-specific ISI and MNPT using e-barcode or analytical calibration with the PT Cal Set (calibrated against National Institute for Biological Standards and Control RBT/05). Before selection of a specific PT-reagent and INR calculation method for future use, we first determined the INR results from 1629 patient samples from the local thrombosis services using both methods and using both INR calculations, and compared these with the INR results of the Hepato-Prest reagent on the STA-R Evolution. The combination of the PT-Owren reagent with INR calculation using the PT Cal set corresponded best with the Hepato-Prest reagent (data not shown) and was therefore selected for all subsequent experiments.

### 2.2 Reproducibility

The reproducibility (i.e. intermediate precision or within-lab variation) was determined by measuring 2 or 3 levels of commercial quality control samples (Roche) in duplicate on 20 separate days, except for anti-Xa which was performed in duplicate on 10 days. Between-run precision results were compared with the performance data specified by the manufacturer and with the desirable specification of imprecision based on within- and between-subject biological variation according to Westgard [10,11].

### 2.3 Method comparison

For this experiment, only excess material of pre-existing and anonymized patient samples collected for routine clinical care was used. Results were used for analytical evaluation only and not reported in the patient file, so patient-informed consent was not required. Samples were collected via venipuncture into evacuated blood tubes containing 0.109 mol/L (3.2%) sodium citrate (BD Vacutainer, Becton Dickinson and Company, Franklin Lakes, NJ) and were measured directly on both the t711 and STA-R Evolution. For anti-Xa measurements, samples from dialysis patients anticoagulated with LMWH or UFH were centrifuged twice to obtain platelet poor plasma (platelet count <10 × 10^9^/L) and immediately frozen until analysis. Because anti-Xa was previously not performed in our laboratory, results from the anti-Xa assay on the t711 were compared with the anti-Xa results determined by a reference laboratory (Radboud University Medical Centre, Nijmegen, the Netherlands) using the STA-R Evolution. The number of samples and the investigated range for each assay is included in Table 3. Data were analyzed using the Passing-Bablok regression method [3,12] in Analyze-it (Analyze-it Software Ltd, Leeds, UK).

### 2.4 Sensitivity to unfractionated heparin

After verification of the anti-Xa assay on the t711, the dose-response of the aPTT assay to UFH was investigated in 41 samples from patients anticoagulated with UFH, in order to determine whether aPTT results can be used to monitor UFH therapy.

The dose-response curve of the aPTT versus anti-Xa activity was empirically fitted using an exponential function in Microsoft Excel 2016, which resulted in a better correlation coefficient than a linear fit.

### 2.5 Sample centrifugation

The standard centrifugation protocol of 15 min at 2500×g as recommended by the manufacturer, was compared with centrifugation for 5 min at 2750×g. For this experiment, two citrated blood samples were obtained from 10 healthy volunteers and from 20 patients treated with vitamin K antagonists. In all samples, platelet count, aPTT, PT, INR, fibrinogen and d-dimer were measured. Anti-Xa was not investigated in this experiment, as a double spin to obtain platelet poor plasma is always required for this assay in order to avoid heparin neutralization by platelet factor 4. Data from both centrifugation protocols were compared using a paired *t*-test in Analyze-it.

### 2.6 Stability in plasma and whole blood

To simulate a delay in sample processing, for instance due to transportation, whole citrated blood samples were drawn from five healthy volunteers and incubated for 0, 2, 4 and 6 h at room temperature. After each incubation step, samples were centrifuged and the aPTT, PT, INR, fibrinogen and d-dimer were determined. Stability of anti-Xa was not investigated since in our hospital anti-Xa activity is always determined within 1 h after blood collection. Similarly, in order to determine the stability in plasma, the citrated plasma samples from the same volunteers were incubated for 0, 2, 4, 6 and 8 h at room temperature. Data were analyzed in Analyze-it using a Dunnett comparison of the average at each time point versus the average at t = 0.

## 3 Results

### 3.1 Reproducibility

Table 2 shows the reproducibility of each assay for the different levels of quality control samples. Results are compared to the specification of performance provided by the manufacturer and to the desired imprecision based on biological variation derived from the Westgard database [10,11]. For aPTT, PT, INR, d-dimer and fibrinogen, the reproducibility was comparable to the manufacturer’s specifications. Additionally, results for all assays easily met the desired imprecision based on biological variation for all these assays, which is an important criterion for clinical application. For anti-Xa, no information on biological variation was available and the specifications claimed by the manufacturer were only met for LWMH. No explanation was found for the higher CV% of the anti-Xa for UFH measured in the current study.

**Table 2:** Overview of the measured and claimed total reproducibility of six coagulation assays on the Roche Cobas t711. Measured coefficients of variation (CV) greater than stated by the manufacturer are highlighted in bold. Required specification for imprecision based on biological variation (I_bio_) is also shown for comparison.

Assay	n	Measured			Claimed			I_bio_ (%)
		Mean	SD	CV(%)	Mean	SD	CV(%)	
aPTT (s)	40	25.5	0.15	0.6	27.2	0.16	0.6	1.4
	40	45.6	0.20	**0.4**	51.5	0.17	0.3	
	40	60.5	0.30	0.5	72.5	0.38	0.5	
PT (s)	40	8.71	0.07	**0.8**	8.96	0.06	0.7	2.0
	40	27.2	0.27	1.0	28.5	0.41	1.4	
INR	40	0.97	0.01	**1.2**	1.03	0.007	0.7	2.0
	40	2.18	0.02	**1.0**	2.05	0.002	0.9	
D-dimer (μg/L)	40	883	8.65	0.98	862	12.7	1.5	11.6
	40	3677	20.5	0.56	3760	33.8	0.9	
Fibrinogen (g/L)	40	1.19	0.02	1.6	1.23	0.02	1.6	5.4
	40	2.82	0.09	**3.2**	2.76	0.06	2.2	
Anti-Xa LMWH (IU/mL)	20	0.39	0.01	2.6	0.355	0.015	4.4	NA
	20	0.94	0.02	1.9	0.930	0.023	2.4	
Anti-Xa UFH (IU/mL)	20	0.25	0.01	**5.7**	0.262	0.013	5.0	NA
	20	0.59	0.02	**3.7**	0.613	0.017	2.8	

aPTT: activated partial thromboplastin time. PT: prothrombin time. INR: international normalized ratio. SD: standard deviation. CV: coefficient of variation. LMWH: low molecular weight heparin. UFH: unfractionated heparin. NA: not available. n: number of replicate measurements.

Our results for reproducibility were similar to previous studies. In a study by Kitchen et al. analytical performance was determined on the Cobas t711 for PT-rec, aPTT and PT-Owren [4]. Although they determined within-run and not between-run reproducibility, their results for the CV of PT-rec (0.2–0.7%), PT-Owren (0.3–0.8%) and aPTT (0.3–1.2%) were comparable to our results and well within the desired imprecision (based on biological variation). Additionally, our results were comparable to the study performed by Lippi et al. [7], who described between-run CV’s of 0.6% and 0.5% for PT-rec at 7.97 s and 27.8 s, and 1.7% and 1.5% for aPTT at 25.8 s and 40.8 s, respectively.

For the D-dimer and fibrinogen assays, our results also compared well to studies performed by Salvagno et al. and Lippi et al. [6,7]. Salvagno et al. determined between-run CV’s for D-dimer of 3.0%, 0.7% and 0.6% at 350 μg/L, 1348 μg/L and, 8026 μg/L, respectively, whereas our results (for 2 levels) were 1.0% and 0.6% at 883 μg/L and 3677 μg/L. For fibrinogen, our results of 1.6% and 3.2% at 1.19 g/L and 2.82 g/L compared well to the results from Lippi et al. [7] of 1.7% and 2.0% at 1.21 g/L and 2.56 g/L, respectively.

### 3.2 Method comparison

Table 3 and Supplemental Figs. 1 and 2 show the results of the method comparison studies between the t711 and the STA-R Evolution. Correlation coefficients varied from 0.92 to 0.99 and were comparable to (or higher than) determined in previous studies for PT (r = 0.97), aPTT (r = 0.81) and fibrinogen (r = 0.97)^7^. Patient results for both the aPTT and PT were significantly lower on the t711 compared with the STA-R Evolution. This was also found in a previous study and can be explained by the differences in coagulation activators and detection methods (Table 1) [7]. Bland-Altman plots showed a consistent difference between the t711 and the STA-R Evolution for the PT and aPTT assays (Supplemental Fig. 2a/b). Therefore, before routine utilization of the t711, clinicians should be well informed about the lower aPTT and PT results and corresponding reference ranges [4].

**Table 3:** Results of the method comparison experiments. The 95% confidence intervals for slope and intercept were determined using Passing-Bablok regression and are shown in parentheses. Statistically significant differences between the Roche Cobas t711 and Stago STA-R Evolution are highlighted in bold (i.e. a slope different from 1 and/or an intercept different from 0).

	n	Range^a^	Slope	Intercept	r
aPTT (s)	89	21.7–131.4	**0.90 (0.80**–**0.98)**	−1.05 (−3.82–1.88)	0.92
PT (s)	83	12.0–43.2	0.85 (0.69–1.04)	**−2.99 (-5.56–-0.79)**	0.93
INR	1629^b^	0.9–6.8	**0.89 (0.88**–**0.90)**	**0.11 (0.09**–**0.13)**	0.98
D-dimer (μg/L)	47	170–6830	1.02 (0.82–1.13)	−3.0 (-115–51)	0.95
Fibrinogen (g/L)	23	2.5–6.5	0.96 (0.85–1.09)	0.07 (−0.38–0.41)	0.95
Anti-Xa (IU/mL)	44^c^	<0.1–1.43	0.99 (0.93–1.06)	0.0005 (−0.02–0.05)	0.99

aPTT: activated partial thromboplastin time. PT: prothrombin time. INR: international normalized ratio. n: number of investigated samples. r: correlation coefficient. LMWH: low molecular weight heparin. UFH: unfractionated heparin.

a Range of results as determined on the STA-R Evolution.

b large number due to inclusion of samples from the local thrombosis services.

c Comprising 24 patients anticoagulated with LWMH and 20 with UFH.

INR-values derived from the PT-Owren on the t711 were on average 6% lower than Hepatoprest values over the entire range, despite international normalization using the INR calibration set to determine the ISI (of 0.904) and MNPT (20.2 s) for this lot. This was also reflected by a negative bias in the Bland-Altman plot (Supplemental Fig. 2c). This difference was also observed in a previous study, where the Owren PT assay on the Cobas t711 was also compared to the HepatoPrest assay on the STA-R MAX [4]. A slope of 0.853 and an intercept of 0.132 with a correlation of 0.994 was found, reflecting a good correlation but significantly lower INR values of the Owren PT on the t711.

To determine the relevance of these lower INR values, the performance of the INR was further investigated by measuring 17 proficiency testing samples with INR-values ranging from 0.99 to 3.40. INR results from the t711 were consistently lower than the mean consensus value for 14 samples, but all results were within the acceptable analytical range (i.e. mean ± 2 × standard deviation; data not shown). The INR was therefore approved for clinical use and thrombosis specialists were informed on the assay differences to allow adequate dosing of vitamin K antagonists.

For d-dimer, fibrinogen and anti-Xa, no significant differences or biases were found. Unfortunately, low fibrinogen levels could not be included because patient samples with decreased fibrinogen were unavailable during the evaluation period. As an alternative, we used six samples from a proficiency testing program with low fibrinogen levels (<2 g/L). This showed excellent agreement (data not shown) and the t711 fibrinogen was therefore approved for the low range samples as well.

### 3.3 Sensitivity to unfractionated heparin

**Fig. 1:**
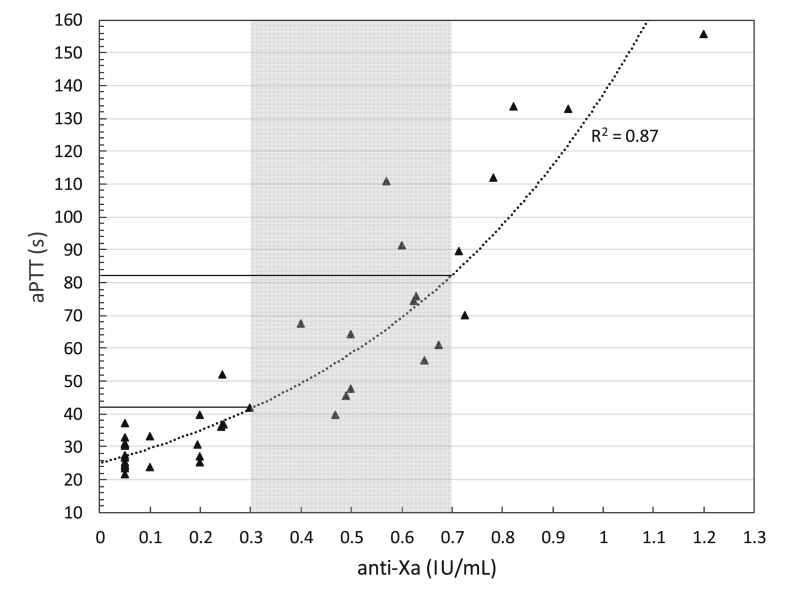
aPTT versus anti-Xa activity as determined in samples from patients receiving UFH therapy. The grey shaded area corresponds with an anti-Xa of 0.3–0.7 IU/mL. The corresponding aPTT therapeutic window is indicated by the horizontal lines.

Fig. 1 shows the dose-response curve of the t711 aPTT versus anti-Xa activity of unfractionated heparin measured in 41 patient samples. A good correlation with an R [2]-value of 0.87 was obtained. The aPTT therapeutic window was subsequently determined by calculating the aPTT corresponding with anti-Xa activities of 0.3 and 0.7 U/mL, which is the common therapeutic range [13]. This resulted in a calculated aPTT range of 42–82 s, which we considered a sufficient window for UFH monitoring.

Interestingly, Kitchen et al. found a considerably more narrow therapeutic window of 46–57 s, which was determined using platelet-poor plasma from 117 patients [4]. A possible explanation is that Kitchen et al. applied a linear fit of their data, resulting in a moderate correlation coefficient of 0.41 (corresponding to an R^2^ of 0.17). In contrast, the R^2^ of 0.87 obtained using an exponential fit, indicates that our model describes the relationship between anti-Xa and aPTT more accurately, even though fewer samples were included.

### 3.4 Sample centrifugation

Table 4 shows the results for the standard and faster centrifugation protocol. Although the number of platelets was significantly higher using the faster centrifugation, no significant differences were found for PT, INR, fibrinogen and d-dimer between both centrifugation protocols. Although aPTT results were on average 0.74 s higher using the standard centrifugation protocol, this difference is clinically irrelevant. The faster sample centrifugation of 5 min at 2750×g is therefore considered suitable for all routine coagulation assays on the t711. This is important for the optimization of sample throughput and allows for a clinically relevant reduction of the turn-around time.

**Table 4:** Average platelet counts and results of the coagulation tests obtained in paired samples centrifuged for 15 min at 2500×g or 5 min at 2750×g. Results are presented as mean ± standard deviation. P-values <0.05 as determined using a paired *t*-test are considered statistically significant. For d-dimer, results are shown for 10 samples only, as the other 20 samples had d-dimer levels below the limit of detection.

	15′ at 2500×g	5′ at 2750×g	P-value
Platelets (x10^9^/L)	5 ± 4	28 ± 17	<0.0001
aPTT (s)	33.5 ± 7.1	32.8 ± 6.6	0.0007
PT (s)	18.0 ± 9.3	17.9 ± 9.3	0.31
INR	1.9 ± 0.8	1.9 ± 0.8	0.18
Fibrinogen (g/L)	3.36 ± 0.75	3.40 ± 0.78	0.11
D-dimer (μg/L)	752 ± 971	715 ± 964	0.24

### 3.5 Stability in plasma and whole blood

Supplemental Figure 3 shows the stability at room temperature for the aPTT, PT, INR and fibrinogen on the t711 in whole blood and plasma obtained from five healthy volunteers. Only one volunteer had detectable d-dimer levels, thereby hampering further analysis. Differences compared with t = 0 were not statistically significant for all other parameters at all investigated time points.

These stability studies showed that additional coagulation tests on the t711 can be safely performed in plasma until 8 h after centrifugation. When sample transportation is required (e.g. from an outpatient clinic to a central laboratory), a delay of maximally 6 h between venipuncture and centrifugation is acceptable. A drawback of the current study is that the experiments were performed on samples obtained from healthy volunteers and that no patient samples were included. Additional stability studies using patient samples with abnormal results are required for complete evaluation.

## 4 Conclusions

The Roche Cobas t711 coagulation analyzer is a user-friendly instrument suitable for routine application in 24/7 clinical laboratories. All investigated assays showed adequate analytical performance in the reproducibility and method comparison experiments. No implications for patient diagnosis and monitoring were experienced upon implementation of the t711 in routine clinical care. Additionally, we found that whole blood and plasma samples are stable for up to 6 and 8 h at room temperature, respectively. This is important for transportation (e.g. from outpatients or from general practice) or when additional coagulation tests are requested in stored plasma. The fast centrifugation protocol of 5’ at 2750×g was found suitable for all routine tests on the t711, thereby allowing improved sample throughput and reduced turn-around times.

Additional experiments to verify potential interferences from hemolysis, icterus or lipemia should be conducted for the t711, since all assays are based on optical detection methods. Furthermore, future studies should include determining the sensitivities of the aPTT and PT to coagulation factor deficiencies and to direct oral factor IIa or factor Xa inhibitors (e.g. dabigatran, rivaroxaban and apixaban).

## Author contributions

MO and JBdK designed the research, analyzed the data and wrote the paper. RLN and JDN performed the research and analyzed the data. All authors drafted or revised the manuscript critically for intellectual content and approved the final version.

## Funding information

This research did not receive any specific grant from funding agencies in the public, commercial, or not-for-profit sectors.

## Declaration of competing interest

The authors have no conflicts of interest to declare.
