# Supplementary material for: Performance of 6 routine coagulation assays on the new Roche Cobas t711 analyzer
Source: Pract Lab Med. 2019 Nov 9;17:e00146. doi: 10.1016/j.plabm.2019.e00146 (PMC6881685; doi:10.1016/j.plabm.2019.e00146)
Supplement: Multimedia component 1 [file mmc1.docx]

**Supporting information to “Performance of 6 routine coagulation assays on the new Roche Cobas t711 analyzer”** **by Oostendorp *et al.***

**Supplemental Figure 1** Passing-Bablok regression analysis of the method comparison experiments for APTT (A), PT (B), INR (C), Fibrinogen (D), D-dimer (E) and anti-Xa (F). Dotted red lines indicate the 95% confidence interval. The identity line is shown in light grey.

**
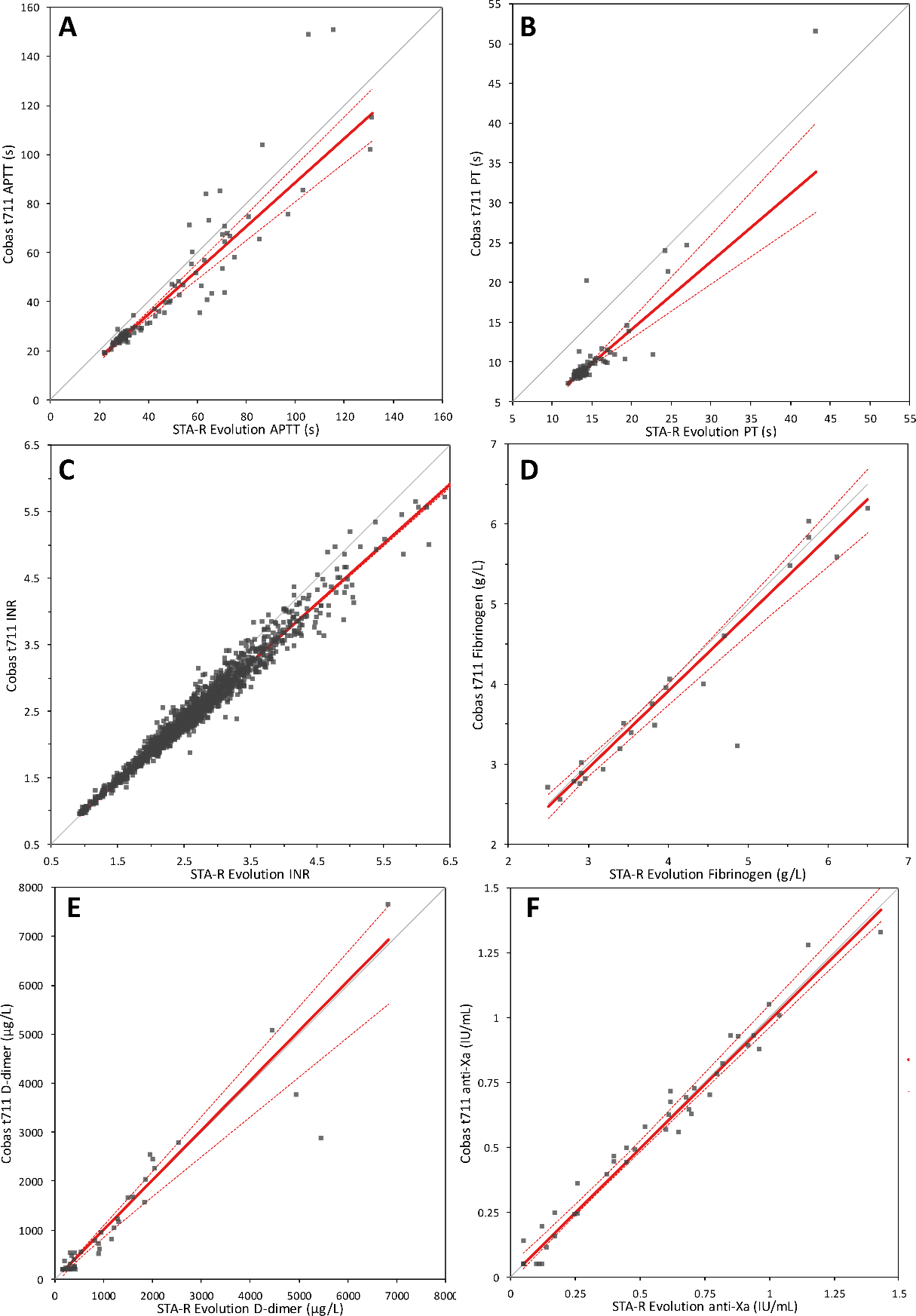
**

**Supplemental Figure 2** Bland-Altman plots for the APTT (A), PT (B), INR (C), Fibrinogen (D), D-dimer (E) and anti-Xa (F). The mean and 95% limits of agreement are shown as the thick blue line and dotted blue lines, respectively.

**Supplemental Figure 2 (continued)**

**Supplemental Figure 3** Stability of the APTT, PT, INR and fibrinogen in whole blood (A) and plasma (B) as determined in 5 healthy volunteers. Data are normalized to the result measured at t=0 (i.e. direct centrifugation and analysis) and are presented as mean ± standard deviation. For all conditions, Dunnett comparison showed no significant differences as compared to t=0.
